# Molecular and Entomological Characterization of 2023 Dengue Outbreak in Dhading District, Central Nepal

**DOI:** 10.3390/v16040594

**Published:** 2024-04-12

**Authors:** Sandesh Rimal, Sabin Shrestha, Sunita Wagle Paudel, Yogendra Shah, Govinda Bhandari, Kishor Pandey, Anjana Kharbuja, Merveille Kapandji, Ishan Gautam, Rajshree Bhujel, Yuki Takamatsu, Rudramani Bhandari, Chonticha Klungthong, Sanjaya Kumar Shrestha, Stefan Fernandez, Gathsaurie Neelika Malavige, Basu Dev Pandey, Takeshi Urano, Kouichi Morita, Mya Myat Ngwe Tun, Shyam Prakash Dumre

**Affiliations:** 1 Central Department of Microbiology, Tribhuvan University, Kathmandu 44601, Nepal; niensandesh@gmail.com (S.R.); sabeinartha@gmail.com (S.S.); kharbujaanjana@gmail.com (A.K.); bhujelrajshree@gmail.com (R.B.); 2 Dhading Hospital, Dhading Besi 45100, Nepal; wsunita2013@gmail.com (S.W.P.); govbhandari@gmail.com (G.B.); rudrabhandari36@gmail.com (R.B.); 3 Seti Provincial Hospital, Dhangadhi 10900, Nepal; yogendra.90@gmail.com; 4 Central Department of Zoology, Tribhuvan University, Kathmandu 44601, Nepal; pandey_kishor@hotmail.com; 5 Department of Virology, Institute of Tropical Medicine, Nagasaki University, Nagasaki 852-8523, Japan; mckkapandji@gmail.com (M.K.); yukiti@nagasaki-u.ac.jp (Y.T.); moritak@nagasaki-u.ac.jp (K.M.); 6 Natural History Museum, Tribhuvan University, Swayambhu, Kathmandu 44620, Nepal; is_gautam@rediffmail.com; 7 Armed Forces Research Institute of Medical Sciences, Bangkok 10400, Thailand; chontichak.fsn@afrims.org (C.K.); sfern012@gmail.com (S.F.); 8 Walter-Reed AFRIMS Research Unit Nepal, Kathmandu 44600, Nepal; shresthask.ca@afrims.org; 9 Faculty of Medical Sciences, University of Sri Jayewardenepura, Colombo 10250, Sri Lanka; neelikamalavige@gmail.com; 10 DEJIMA Infectious Disease Research Alliance, Nagasaki University, Nagasaki 852-8523, Japan; drbasupandey@gmail.com (B.D.P.); turano@med.shimane-u.ac.jp (T.U.); 11 Center for Vaccines and Therapeutic Antibodies for Emerging Infectious Diseases, Shimane University, Izumo 690-8504, Japan; 12 Department of Tropical Viral Vaccine Development, Institute of Tropical Medicine, Nagasaki University, Nagasaki 852-8523, Japan

**Keywords:** dengue outbreak 2023, dengue virus serotypes, entomological investigation, *Aedes* spp., vector density, Nepal

## Abstract

In 2023, Nepal faced its second largest dengue outbreak ever, following a record-breaking number of dengue cases in 2022, characterized by the expansion of infections into areas of higher altitudes. However, the characteristics of the 2023 circulating dengue virus (DENV) and the vector density remain poorly understood. Therefore, we performed DENV serotyping, clinical and laboratory assessment, and entomological analysis of the 2023 outbreak in central Nepal. A total of 396 fever cases in Dhading hospital suspected of being DENV positive were enrolled, and blood samples were collected and tested by different techniques including PCR. Of these, 278 (70.2%) had confirmed DENV infection. Multiple serotypes (DENV-1, -2, and -3) were detected. DENV-2 (97.5%) re-emerged after six years in Dhading while DENV-3 was identified for the first time. Dengue inpatients had significantly higher frequency of anorexia, myalgia, rash, diarrhea, nausea, vomiting, abdominal pain, and thrombocytopenia (*p* < 0.05). In this area, *Aedes* mosquitoes largely predominated (90.7%) with the majority being *A. aegypti* (60.7%). We also found high levels of *Aedes* index (20.0%) and container index (16.7%). We confirmed multiple DENV serotype circulation with serotype re-emergence and new serotype introduction, and high vector density in 2023. These findings call for the urgent initiation and scaling up of DENV molecular surveillance in human and mosquito populations for dengue control and prevention in Nepal.

## 1. Introduction

Dengue is caused by infection with any of the four dengue virus (DENV) serotypes (DENV-1, -2, -3, and -4) and transmitted through bites of female mosquitoes (*Aedes aegypti* and *A. albopictus)* [1,2,3]. Due to rapid unplanned urbanization, dengue has been an emerging threat with a drastic increase in incidence in the recent years (>300% since 2009). As a consequence, 3.9 billion people living in 128 countries are at risk of dengue, with 96 million apparent infections reported annually, and the majority of cases are from the South Asia region [4,5,6]. In the past few decades, DENV has spread throughout this region, causing annual epidemics of increasing magnitude in Bhutan, India, Maldives, Bangladesh, Sri Lanka, Pakistan, and Nepal [2]. In 2023, neighboring Bangladesh was severely hit by a dengue outbreak with dominant serotypes 2 and 3, resulting in a high number of fatalities [7].

Dengue was first detected in Nepal in 2004 [8,9], but all four DENV serotypes were identified in the first outbreak in 2006 [10]. Since 2010, significant outbreaks occurred in the years 2013, 2016, 2019, and a massive outbreak in 2022 with 54,784 cases and 88 casualties affecting all 77 districts [11,12,13,14,15]. Serotype(s) responsible for each of these outbreaks varied, indicating a potential DENV serotype switching leading to Nepal’s unique dengue pattern [10,15]. Due to the paucity of molecular data, there is no clear understanding on how the virus serotypes were introduced, and how they established and produced fulminating outbreaks in Nepal. In the past, Nepal has experienced dengue outbreak in a cyclical trend of 2–3 years [15,16,17], however the country has faced consecutive outbreaks of larger magnitude in recent years, including 2022 and 2023. The Nepalese Epidemiology and Disease Control Division (EDCD) reported 51,243 dengue cases in the 2023 outbreak that mainly affected eastern and central Nepal [18]. 

Dengue has been geographically expanding in Nepal, with increasing morbidity and mortality [14,19]. Despite this, very limited entomological studies have been conducted to understand the distribution and abundance of mosquito vectors. *A. albopictus* has a long history in Nepal dating back to the 1950s [20,21], while *A. aegypti* was first reported in 2006 [10,11]. Continued migration and proliferation of these mosquitoes in new breeding habitats have been reported at higher altitudes. *Aedes* was detected at an altitude of 2100 m above mean sea level (MSL) in Rasuwa district in 2014 [22]. Interestingly, these vector trends coincide well with the recent 2022 outbreak, when EDCD reported dengue cases in all 77 districts of Nepal [15,18,23]. Rapid urbanization, vehicular movement, unmanaged solid waste disposal, human migration, an open boarder to India, and environmental changes, like fluctuation in rainfall and rising temperatures, may be key players in the proliferation of mosquitoes in high Himalayan region, which need to be carefully evaluated [7,16,24,25]. Moreover, climate change affects the fitness of the DENV and its vector mosquitoes, and leads to alterations in transmission environment. This eventually fluctuates the rate of dengue incidence and transmission patterns [26,27].

To fully understand the reasons for dengue expansion and the occurrence of larger outbreaks in the country, it is crucial to establish robust a national dengue case and entomological surveillance program to obtain more annual data, particularly regarding case profiles, virus characteristics, vector abundance and competency, and overall climatic factors. Nevertheless, studies on patients, the virus, and vectors in a specific geographical location during an unprecedented outbreak provide valuable insights into the dengue trend. This will also serve as baseline information for scaling up future studies and initiating nationwide surveillance by the dengue control program. Therefore, we carried out assessment of the patient profiles, entomological characteristics, and DENV serotype distribution in the Dhading district, one of the worst-hit areas during the 2023 dengue epidemic in Nepal.

## 2. Materials and Methods

### 2.1. Study Design and Setting

**Figure 1:**
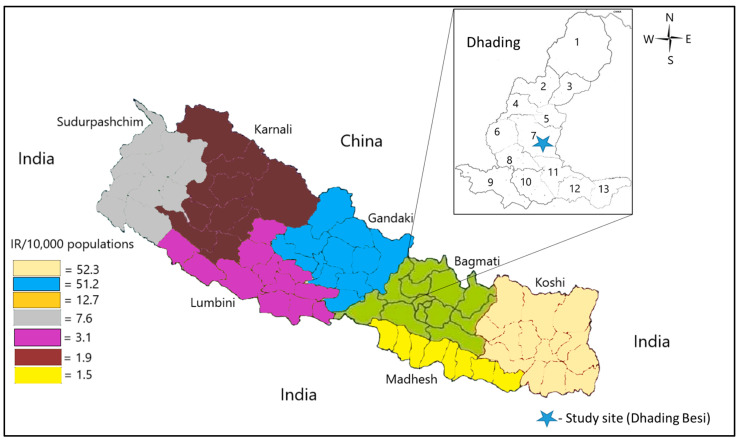
Nepal map showing provincial incidence of dengue and the current study area (Dhading district with its 13 municipalities). 1, Rubivalley; 2, Gangajamuna; 3, Khaniyabash; 4, Tripura Sundari; 5, Netrawati; 6, Jwalamukhi; 7, Nilkantha; 8, Siddhalek; 9, Benighat Rorang; 10, Gajuri; 11, Galchii; 12, Tharke; 13, Dhunibesi. Dengue incidence rate (IR) per 10,000 population in all seven provinces of Nepal is presented with different color codes [18].

This study was conducted in the Dhading district of Bagmati province in central Nepal, as it was among the hardest hit districts by dengue. The district extends from 27°57′04.68″ N Latitude to 85°00′09.36″ E longitude and covers 192,487 hectares in total, with a total population of 322,751 [28]. The district has a very wide range of elevation from 488 m to 7409 m with a sub-tropical to temperate climate. The district is bordered by Kathmandu (Capital city) and Nuwakot in the east, Gorkha in the west, Makawanpur and Chitwan in the south, and Rasuwa district in the north. Additionally, the northern border connects with Tibet of China (Figure 1). The average annual rainfall in Dhading is 2121.2 mm. The eastern region experiences the longest rainy season, whereas the western and northern regions experience a slightly shorter one due to altitudinal variation. In Dhading, June is the warmest month (average 29.4 °C) and January is the coldest month (average 3.7 °C) of the year, but the temperature may vary according to area with a range of 5.35 °C to 35.22 °C [29].

The study hospital (Dhading hospital) is situated in the district headquarters Dhading Besi, which is a small town nestled between two rivers, the Arun Khola and the Thopal Khola. This is a 50-bed public hospital with services in general medicine, gynecology, pediatrics, dermatology, general surgery, orthopedics, neurology, and radiology, along with the diagnosis and treatment of tropical and infectious diseases including dengue.

### 2.2. Human Component

#### 2.2.1. Patient Enrollment, Questionnaire, and Sample Collection

Febrile patients suspected of having dengue, regardless of age or gender, visiting at Dhading hospital were included in the study. A standardized questionnaire was administered to gather demographic data, history of past infection, and travel. A blood sample (3–5 mL) was collected during the acute phase of infection then 3 aliquots of serum and EDTA-blood were prepared. Serum samples were transported to and stored at −80 °C/−60 °C at the Central Department of Microbiology (CDMi) laboratory, Tribhuvan University, Kathmandu, Nepal, until further analysis.

#### 2.2.2. Dengue Diagnosis

Dengue infections were diagnosed by detecting non-structural (NS1) antigen/IgM/IgG antibody using a combo test (Dengue NS1 Ag + Ab, Biotrol laboratories Pvt. Ltd., New-Delhi, India) and/or RT-PCR (Takara Bio Inc., Shiga, Japan) as per the kit manufacturer’s instructions. Dengue cases were classified according to the WHO 2009 dengue guidelines [30]. Additionally, dengue patients were also categorized as those requiring hospitalization and those who recovered as outpatients.

**Figure 2:**
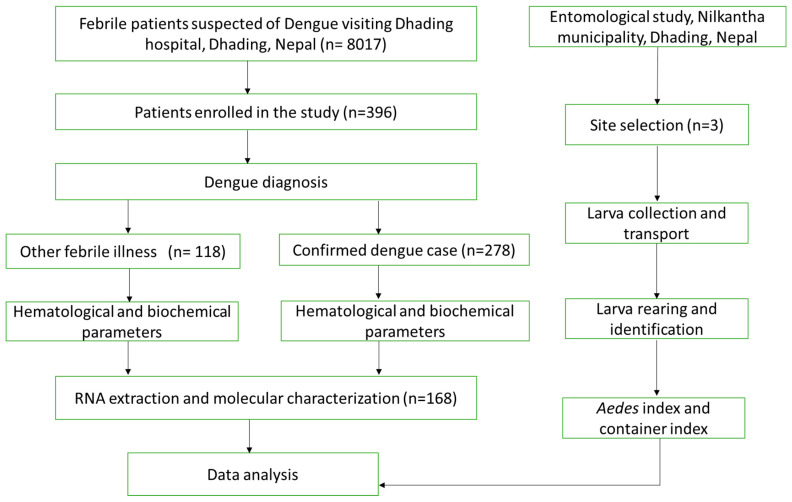
Flow diagram of the study. Out of 8017 febrile patients, 2814 (35.1%) were dengue positive.

Clinical and laboratory data were obtained from medical records and laboratory data management system, respectively. Of the total 396 samples collected, 168 samples from dengue suspected patients were randomly selected and subjected to DENV Reverse Transcription Polymerase Chain Reaction (RT-PCR) (Figure 2).

#### 2.2.3. Viral RNA Extraction

Viral RNA was extracted using the QIAamp Viral RNA kit (QIAGEN, Hilden, Germany) directly from 140 μL of the patient’s serum as per the manufacturer’s manual. Using NanoDrop (Thermo Fisher Scientific, Inc., Waltham, MA, USA), viral RNA was quantified and its quality was evaluated.

#### 2.2.4. One-Step Reverse Transcription Polymerase Chain Reaction (RT-PCR)

RT-PCR was used to identify the presence of DENV RNA using the PrimeScriptTM One Step RT-PCR Kit Ver. 2 (Takara Bio Inc., Shiga, Japan). Briefly, a final volume of 15 μL was used for the RT-PCR amplification, which contained 5 μL of RNA. The RT-PCR reaction mixture contained 0.5 μL of enzyme mix, 7.5 μL of 2× buffer, 1 μL of nuclease-free water, and 0.5 μL of 100 pmol forward and reverse primers, with different primer sets (Supplementary Table S1) for the detection of DENV and the identification of serotypes [15,31,32,33]. The RT-PCR program consisted of the following steps: 42 °C for 60 min; 35 cycles of 94 °C for 30 s; 55 °C for 1 min; 72 °C for 2 min, and was carried out using the BioRad T100 Thermal Cycler (Bio-Rad Laboratories, Hercules, CA, USA). DNase/RNase-free water (Sigma, New York, NY, USA) served as a negative control, while a mixture of all four DENV serotypes (DENV-1 (99St12Astrain, PP422232), DENV-2 (00St22A, PP422234), DENV-3 (SLMC 50 strain, PP425980), and DENV-4 (SLMC 318 strain, PP422236) with a known viral concentration (10^6^ FFU/mL of DENV-1, -3, and -4 and 10^7^ pfu/mL of DENV-2) was used as a positive control [15]. Amplified PCR products were detected by agarose gel electrophoresis and visualized and recorded using the Azure 200 Workhorse Gel Imager (Azure Biosystems Inc., Dublin, CA, USA).

### 2.3. Entomological Component

#### 2.3.1. Site Selection

Nilkantha municipality, the most severely affected area during the 2023 dengue outbreak in Dhading, was selected for entomological investigation. Out of the 14 wards in the Nilkantha municipality, the most affected wards (wards 3, 4, and 6) were surveyed for the distribution and density of mosquito vectors in the month of August based on the indicator cases initially reported in the hospital. The cases from the hospital were closely followed up, and their households were chosen as representative sites for mosquito vector survey. The premises and the areas permitted by the household owner were inspected for mosquito breeding. The presence of immature stages of mosquito was visually evaluated in all water-holding containers located in outdoor areas. Outdoor areas were defined as the outside of the house, but confined to its immediate proximity (i.e., located within 10 m). Altogether five different types of habitats were investigated for mosquito larvae and pupae: discarded tires, drums (plastic and metal drums), plastic containers (plastic pots and plastic buckets), metal containers, and flower pots (mud pots).

#### 2.3.2. Larva/Pupa Collection and Transport

Larval and pupal *Aedes* mosquitoes were collected from each sampling site using the dropper and dipper technique [11,34]. They were collected with a wide-mouthed plastic dropper directly from the water surface in the mosquito habitat or from water samples taken with a dipper [35]. The number and type of containers inspected were recorded, including information on the presence or absence of immatures. Collected larvae were kept in plastic bags with water, and thereafter placed immediately in a labeled icebox and transported to the laboratory of the Central Department of Microbiology, Tribhuvan University, Kirtipur, Kathmandu within 5 h maintaining suitable conditions for larvae.

#### 2.3.3. Larva/Pupa Rearing and Adult Identification

All larval and pupal *Aedes* mosquitoes were reared in the pre-sterilized plastic cups until adults emerged, and they were fed with mixture of dog food and yeast powder [36] when necessary. Adults that emerged from the pupae were collected from the plastic cups using aspirator then flash frozen for morphological identification to species level using standard keys [20,37,38]. Thereafter, adult mosquitoes were stored in cyro-vials at −80 °C for further analysis.

#### 2.3.4. Estimation of Mosquito Distribution and Its Density

The *Aedes* index (AI) was calculated by dividing number of houses infested with *Aedes* spp. by number of houses inspected. Similarly, container index (CI) was calculated by dividing the number of positive containers by number of containers inspected. Both AI and CI were then expressed into percentages using the following formula [34,39].

$$\mathrm{Aedes}\mathrm{index}\;(\mathrm{AI}):\frac{\mathrm{Number}\;\mathrm{of}\;\mathrm{houses}\;\mathrm{infested}}{\mathrm{Number}\;\mathrm{of}\;\mathrm{houses}\;\mathrm{inspected}}\times 100\%\;$$

Container index (CI):Number of positive containersNumber of containers inspected×100% 

### 2.4. Data Analysis

Data were entered into Microsoft Excel sheets, and, after proper cleaning and verification, they were exported to statistical analysis software (SPSS for Windows, version 25.0) (IBM Corp., Armonk, NY, USA). Continuous variables were presented as median (25–75% inter-quartile range (IQR)) and categorical variables as absolute number (n) and percentage (%). The comparison of continuous variables between two groups was performed using the Mann–Whitney U test, whereas the chi-square test (or Fisher’s exact test) as appropriate was used to compare the categorical variables. All tests were considered statistically significant at an alpha error of 0.05. 

## 3. Results

### 3.1. Seasonality Trend of Dengue and Climatic Conditions in Dhading, Nepal, 2023

**Figure 3:**
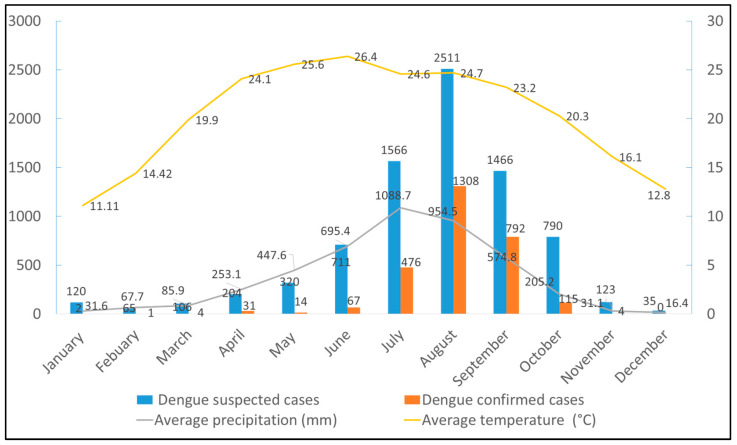
Seasonal trend of dengue and monthly climatic parameters in Dhading, Nepal, 2023.

There was a total of 8017 dengue suspected cases in Dhading hospital, of which 2814 (35.1%) were found to be dengue positive by NS1. During this dengue outbreak, sporadic dengue cases started to appear in Dhading as early as January (n = 2). As the monsoon approached on 13 June, a rapid rise in cases resulted in a peak in August with an exponential trend with 46.5% (1308/2814) of the total cases in Dhading hospital occurring in a single month. Subsequently, the epidemic started to decline in September (n = 792) with zero case in December (Figure 3). 

### 3.2. Demographic, Clinical, and Laboratory Parameters of Dengue Patients

We enrolled 396 febrile patients suspected of having dengue (age range: 2–88 years, median (IQR) age: 31 years (18–45)). Among them, 278 (70.2%) were confirmed dengue cases, while the remaining patients had other febrile illnesses (OFI) of unknown origin(s). Dengue and non-dengue patients differed in age (*p* < 0.001), with the majority of the dengue patients being adults (83.9%). The clinical characteristics significantly associated with dengue illness were retro-orbital pain, rashes, and gastrointestinal symptoms (*p* < 0.05). Moreover, laboratory parameters comprising total leucocyte count, neutrophils count, lymphocytes count, platelets count, and admission requirement, were significantly different between dengue cases and OFI patients (*p* < 0.05). (Supplementary Tables S2 and S3).

Of the 278 dengue patients, 44 patients (15.8%) required hospital admission. Among total dengue cases, adult dengue patients (n = 41) required admission at a greater rate (14.7%) than pediatric patients (1.1%; *p* = 0.046), and the median age for the inpatients and outpatients was 34.5 (27–42) and 31.5 (19.5–43.5) years, respectively. Several clinical features (anorexia, myalgia, rash, diarrhea, nausea, vomiting, persistent vomiting, and abdominal pain) were significantly different between inpatients and outpatients (*p* < 0.05) (Table 1).

Similarly, dengue patients requiring admission had significantly lower platelet count compared to the outpatient group at the time of first hospital visit (*p* < 0.05). Other hematological and biochemical profiles were not different between these groups (Table 2).

### 3.3. Distribution of Circulating DENV Serotypes in Dhading, Nepal, 2023

**Figure 4:**
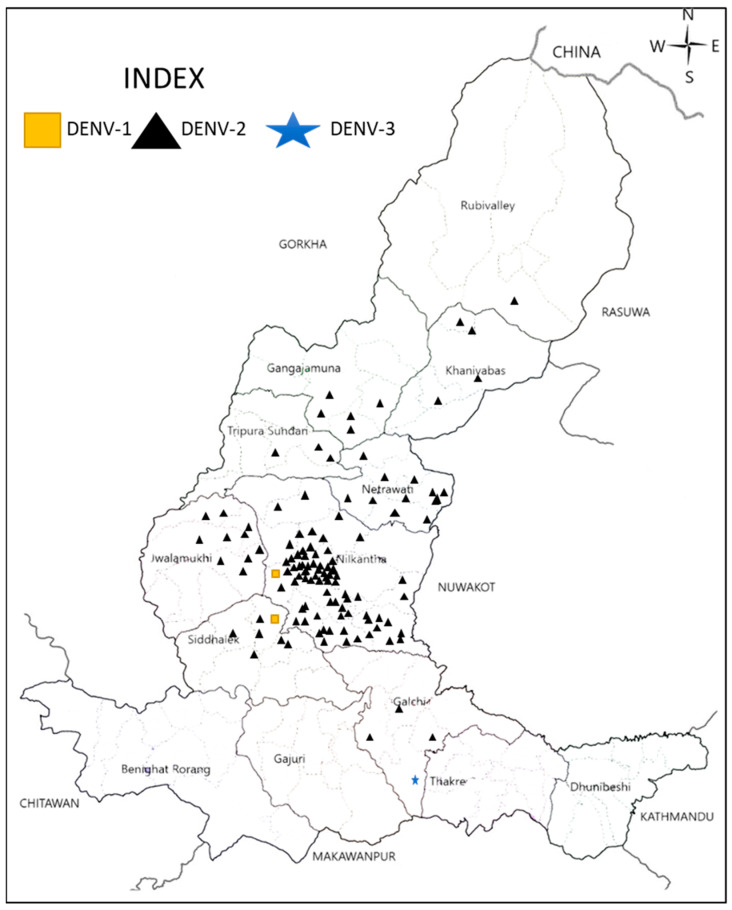
Map of Dhading district showing multiple DENV serotypes circulation in human in 2023.

During the 2023 outbreak in Dhading, we confirmed the circulation of three serotypes of DENV (DENV-1, -2, and -3), but not DENV-4 from dengue patients (Figure 4). A total of 168 serum samples from DENV suspected patients were selected for further molecular analysis. Of these, 120 (71.43%) were found to be DENV positive by RT-PCR.

DENV-2 (n = 117, 97.5%) was responsible for the 2023 outbreak in Dhading with sporadic detection of DENV-1 (n = 2, 1.67%) and DENV-3 (n = 1, 0.8%). Nilkantha, Siddhalek, and Galchi municipalities were found to have multiple serotypes (DENV-1 and 2 in Nilkantha and Siddhalek while DENV-2 and-3 in Galchi), whereas only DENV-2 was detected in other municipalities (Figure 4). 

**Figure 5:**
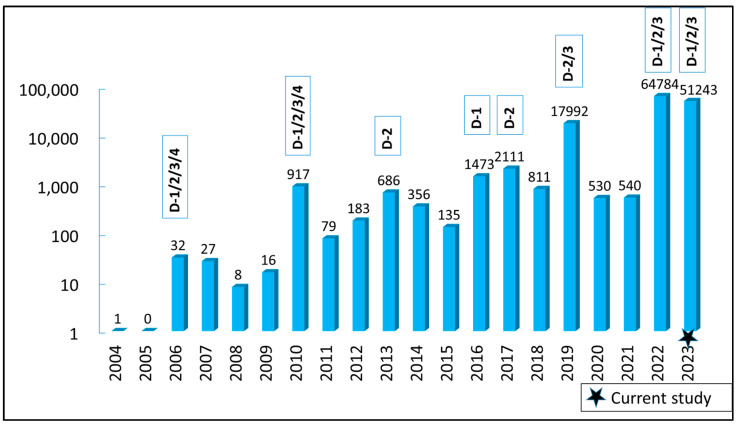
Trend of dengue cases in Nepal with available DENV serotype distribution during 2004 to 2023 [8,10,11,12,13,14,15,18,19,40,41,42]. DENV serotypes are given in the box above the bar for each year with data availability. Y-axis indicates the number of cases in log-scale and X-axis denotes year.

On a national level, dengue in Nepal has been exponentially increasing since it first appeared in the country [11,14,15,18,40,41]. With the increasing trend of dengue cases in Nepal, serotype switching phenomenon among all 4 serotypes was also observed during the major outbreaks occurred between 2004 and 2023 (Figure 5). 

### 3.4. Mosquito Vector Distribution in the Selected Areas of Dhading during 2023 Dengue Outbreak

**Figure 6:**
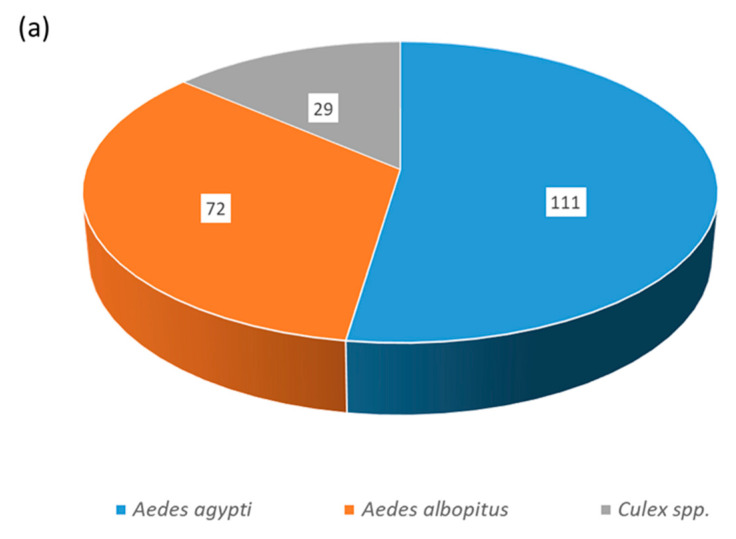

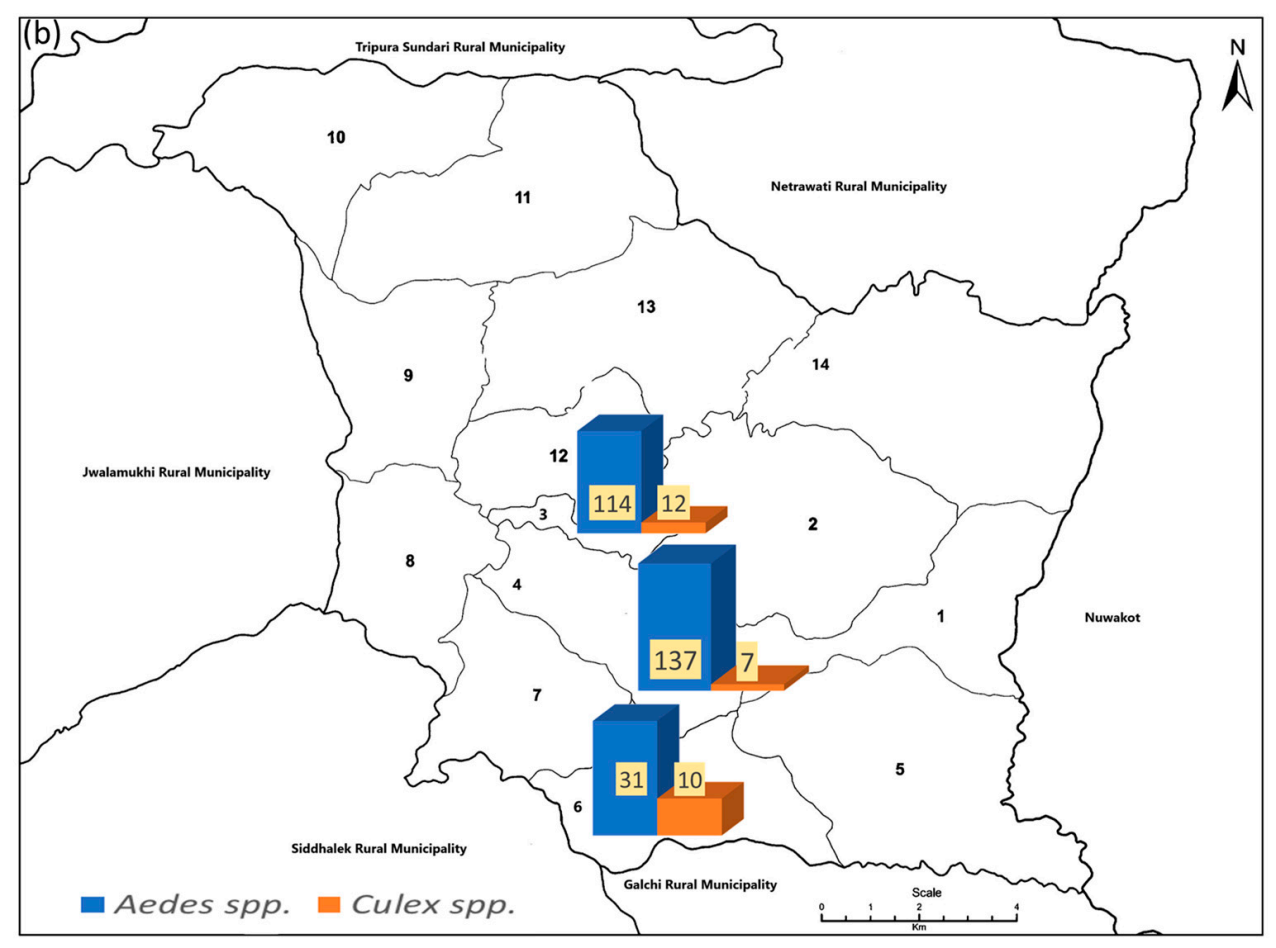
Density of mosquito vectors in Dhading during 2023 dengue outbreak. (**a**) Mosquito density by species. (**b**) Ward-level map of Nilkantha municipality showing vector density in the bar diagrams for each of 3 wards (wards 3, 4, and 6) that underwent entomological investigation. Numbers (1 to 14) in the map indicate ward numbers (sub-municipality divisions) of Nilkantha municipality.

Out of the 311 mosquitoes collected around the study area, 90.7% (282/311) were *Aedes* spp. with 64.9% (183/282) female, and 9.3% (29/311) were *Culex* species. Most of the *Aedes* females (60.7%, 111/183) were *A. agypti*, while the remaining 39.4% (72/183) were *A. albopictus* (Figure 6).

### 3.5. Mosquito Density by Its Habitat

The potential breeding habitats were found to be discarded tires, drums, plastic containers, and flowerpots. A total of 100 households and 126 containers were inspected for the presence of mosquito larvae and pupae. Of which, 20 households and 21 containers had mosquito larvae. The overall AI and CI were 20.0% and 16.7%, respectively (Table 3). High vector density was observed in wards 3 and 4, which were the major hotspots of dengue during the 2023 outbreak in Dhading. 

## 4. Discussion

Dengue has emerged as a significant public health threat in Nepal, due to an exponential increase in cases and its expansion to higher altitudes (temperate to sub-alpine climate) and territories [18]. Over the past five years, the country has encountered three big outbreaks in 2019, 2022, and 2023 with 17,992, 64,784, and 51,243 confirmed dengue cases, respectively [15,18,43,44]. Dhading became the fourth hardest hit district, with 6.3% of all cases in Nepal during the 2023 outbreak [18]. DENV-2 was responsible for this large outbreak in Dhading in 2023, since the vast majority belonged to this serotype, although DENV-1 and DENV-3 were also detected. Though it has only been two decades since Nepal reported the first ever dengue case, major dengue outbreaks have occurred periodically in 2010, 2013, 2016, 2017, 2019, and 2022 with predominant serotypes DENV-1/-2, DENV-2, DENV-1, DENV-2, DENV-2/-3, DENV-1/-3, respectively (Figure 5) [13,14,15,42,45]. The available information confirms the constant circulation of multiple DENV serotypes and also suggests a serotype switching phenomenon in Nepal. In 2006 and 2010, there were reports of all four DENV serotypes [10,42]. However, there has been no report of DENV-4 in Nepal after 2010. It is not conclusive whether DENV-4 has been truly absent there for several years now, or a lack of systematic surveillance has failed to capture the relatively rare serotypes in the country. 

Only limited data on DENV serotype information are available from different outbreaks in Nepal, which hinders the understanding of the real-world scenario. Furthermore, this holds true in the context of Dhading too while looking at the historical data to link the dengue outbreaks. DENV-1 and DENV-2 remained the cause of dengue outbreaks in Dhading at different time points: a case of DENV-2 in 2006 [10] and a couple of DENV-2 cases in 2017 [19], DENV-1 in 2010 [11], and mainly DENV-1 in 2022 [15]. It can be speculated that the population was probably naïve to DENV-2 as only a few DENV-2 cases were reported earlier in Dhading. Although all of these studies had sampling limitations which likely underestimate the actual serotype distribution, it is clear that DENV-2 is well-sustained in Dhading since its reintroduction in 2017 after its absence for 11-year. In an area with multiple serotype circulation like Dhading, relative prevalence levels of each serotype are characterized by an oscillatory behavior with periods of 8–10 years [46,47]. Being a neighboring district of the capital city, Kathmandu, with the highest urban population in the country, the risk of a spill-over of DENV-2 from Dhading to Kathmandu might occur anytime in the future and may cause an unprecedented epidemic. Albeit few in numbers, this is the first time that DENV-3 was introduced in the area but did not cause a large outbreak. However, such introduction of a new serotype may favor a larger dengue outbreak in future [48]. The serotype displacement phenomenon perhaps had a crucial impact on the historically biggest dengue epidemic which occurred in 2022 in Nepal, though the serotype data for each year is not available [15,23,49]. Clade or genotype shifts may also contribute to the rapid surge in dengue cases, as seen in the neighboring country Bangladesh during the 2017 outbreak [50]. Interestingly, Bangladesh also had a severe dengue outbreak in 2023 with increased fatality rates, indicating a regional concern in South Asia [7]. Further investigations into antigenic changes of clade or genotype circulation over time in Nepal may provide better insights into the relationship between DENV clade or genotype changes and evolution of outbreaks.

An abundance of *Aedes* vectors (*A. aegypti* as major species) was observed in the study area with a high vector density around the hardest hit areas in Dhading. Interestingly, *A. agypti* was also reported in Dhading in 2010 [11]. The species pattern is similar to the previous entomological findings from Nepal [22]. We observed higher AI and CI in Dhading than in the previous report from Nepal [51]. The presence of both species of *Aedes* vectors, their high density around the hotspots, and probably DENV-2 specific non-immune population in Dhading favored such a large-scale dengue outbreak in 2023. Previous findings also support this hypothesis, since a positive correlation exists between vector density and burden of dengue [22,52]. Moreover, population dynamics of *Aedes* mosquitoes and their density are strongly dependent on climatic conditions [53,54]. Although there are no data to explain factors that caused high vector proliferation in Dhading during this outbreak, the impact of climate change, virus traits, unplanned and rapid urbanization, waste disposal problems and poorly maintained drainage systems, and increased trade, vehicular transportation, and tourism, likely contributed to vector population and subsequently erupted into an outbreak [16,53,54,55,56]. Nonetheless, dengue incidence dynamics are thought to be driven by a complex interplay of such local factors [57,58].

The 2023 dengue outbreak in Dhading showed a seasonality peaking in August which used to be September in the previous years [11,12,15,40,42]. This changing pattern may be correlated with climatic factors, including global warming, as the year 2023 experienced a rise in the average maximum temperature by 0.6 °C than the previous year [59,60]. Similarly, temperature, precipitation, and relative humidity were at their maximum as early as July [29], which might have provided the favorable conditions for mosquito breeding and proliferation (Figure 3). Temperature and precipitation are key factors in a mosquito’s life cycle, and are greatly affected by climate change [56,61]. Such an increase in mosquito density and temperature are critical for dengue outbreak in the future [58]. Moreover, in the upcoming decades, mosquito abundance is expected to increase [56,61], and the worst dengue epidemic with fatalities is yet to come. In this situation, the effective dengue vaccine is the most promising approach to tackle this disease, as there are no specific antivirals and 100% effective vector control strategies [62,63].

The number of dengue cases in Dhading has been exponentially rising (sporadic in 2010, 7 in 2017, 131 in 2019, 1683 in 2022, and 3239 in 2023) [11,15,18,40,41]. Dhading hospital alone reported two deaths due to dengue in 2023, although the majority of severe patients and those likely to progress to severe dengue are often referred to higher health facilities in the capital city due to its close proximity. A relatively lower rate of hospitalization among children than adults might be attributable to the higher priority given to children for treatment in better health facilities in the capital city rather than in district hospitals. This might be the reason behind a very small number of severe dengue cases for which a precise severity analyses could not be accomplished. This was a limitation of this study. Despite this, several clinical and laboratory features were found to be different between inpatients and outpatients. This information might be helpful in patient management as well as to evaluate compliance with dengue guidelines in future outbreaks, especially in the peripheral hospitals. 

## 5. Conclusions

We confirmed the circulation of three DENV serotypes during the 2023 outbreak in Dhading, Nepal, with a predominance (97.5%) of DENV-2 which was re-reintroduced in Dhading in 2017 with only a handful of cases. After six years, in 2023, DENV-2 caused a big outbreak in Dhading. Moreover, we also identified DENV-3 for the first time in Dhading, indicating the potential for another outbreak due to the non-immune population to DENV-3. High density of *Aedes* vectors were found in the hotspots of the dengue outbreak in Dhading. Molecular profiling of DENV and entomological data on the occurrence and ranges of vectors are crucial for determining the likelihood of outbreaks and informing early warning systems, which, in turn help in early interventional and preparedness activities. Given the increased risk of dengue including in the high-altitude regions, initiating molecular surveillance and sequencing of DENV strains from both field-collected *Aedes* mosquitoes and the human population should be the priority for control programs and research.

## Figures and Tables

**Table 1:** Demographic and clinical characteristics of dengue patients, Dhading, Nepal, 2023.

Characteristics	Category	Outpatients	Inpatients	*p*-Value
Age	Child	45 (93.7)	3 (6.3)	<0.001
	Adult	189 (82.2)	41 (17.8)	
Gender	Female	126 (82.4)	27 (17.6)	0.303
	Male	112 (86.4)	17 (13.2)	
Travel history	No	52 (65.0)	28 (35.0)	0.145
	Yes	3 (37.5)	5 (62.5)	
Myalgia	No	22 (84.6)	4 (15.4)	0.007
	Yes	33 (53.2)	29 (46.8)	
Rash	No	43 (72.9)	16 (27.1)	0.004
	Yes	12(41.4)	17 (58.6)	
Vomiting	No	50 (84.7)	9 (15.3)	<0.001
	Yes	5 (17.2)	24 (82.8)	
Persistent	No	55 (77.5)	16 (22.5)	<0.001
vomiting	Yes	0 (0.0)	17 (100.0)	
Nausea	No	38 (90.5)	4 (9.5)	<0.001
	Yes	17 (37.0)	29 (63.0)	
Retro-orbital pain	No	24 (66.7)	12 (33.3)	0.502
	Yes	31 (59.6)	21 (40.4)	
Any bleeding	No	55 (62.5)	33 (37.5)	-
Anorexia	No	25 (78.1)	7 (21.9)	0.022
	Yes	30 (53.6)	26 (46.4)	
Diarrhea	No	53 (67.9)	25 (32.1)	0.005
	Yes	2 (20.0)	8 (80.0)	
Abdominal pain	No	55 (72.4)	21 (30.5)	<0.001
	Yes	0 (0.0)	12 (100.0)	

The chi-square test was used to analyze categorical variables. Figures in the parentheses indicate percentages.

**Table 2:** Hematological and biochemical profiles of dengue patients, Dhading, Nepal, 2023.

Blood Parameters	Outpatient, Median (IQR)	Inpatient, Median (IQR)	*p*-Value
Hemoglobin	12.8 (11.7–14.3)	12.6 (11.4–14.3)	0.579
Total count (WBC)	5280 (3785–6530)	4945 (2820–6705)	0.142
Neutrophils	67.5 (56.0–75.0)	69.5 (57.3–75.0)	0.414
Lymphocytes	21.0 (16.0–31.0)	21.5 (14.0–29.0)	0.579
Eosinophils	1.0 (1.0–3.0)	1.5 (1.0–2.0)	0.648
Monocytes	8.0 (5.0–11.0)	7.5 (5.0–11.0)	0.518
Platelets	173.5 (127.3–238.5)	138.0 (54.0–174.0)	<0.001
HCT	40.3 (36.5–44.3)	40.6 (36.2–44.2)	0.838
Serum urea	41.0 (33.0–50.5)	42.0 (28.0–57.0)	0.704
Creatinine	1.0 (0.9–1.1)	1.0 (0.9–1.1)	0.708
Sodium	136.0 (134.0–138.0)	137.0 (136.0–139.0)	0.107
Potassium	3.8 (3.5–4.0)	3.8 (3.2–4.0)	0.651
SGPT	46.0 (23.5–67.0)	32.5 (25.8–55.5)	0.276
SGOT	42.0 (31.5–83.5)	34.5 (25.3–51.8)	0.271
ALP	234.0 (185.0–316.0)	245.0 (196.0–342.0)	0.252
Bilirubin—total	0.6 (0.6–0.8)	0.6 (0.6–0.7)	0.459
Bilirubin—direct	0.2 (0.1–0.2)	0.2 (0.1–0.2)	0.122

The Mann–Whitney U test was used to compare continuous variables between two groups. IQR, inter-quartile range; WBC, white blood cells; HCT, hematocrit; SGPT, alanine aminotransferase; SGOT, aspartate aminotransferase; and ALP, alkaline phosphatase.

**Table 3:** Distribution of mosquito vectors according to their habitats in dengue hotspots in Dhading, Nepal during the 2023 outbreak.

Location	Number of Households Inspected	Types of Habitats	Number of Containers with Larvae (%)	Identified Mosquitoes
Nilkantha-3	30	Tires (n = 13)	3 (23.1)	*Aedes* spp.
		Drums (n = 12)	0 (0.0)	
		Plastic containers (n = 8)	3 (37.5)	*Aedes* spp.; *Culex* spp.
		Metal container (n = 6)	0 (0.0)	
Nilkantha-4	40	Tires (n = 4)	0 (0.0)	
		Drums (n = 16)	2 (12.5)	*Culex* spp.; *Aedes* spp.
		Plastic Containers (n = 10)	2 (20.0)	*Aedes* spp.
		Metal container (n = 7)	0 (0.0)	
		Garbage containers (n = 6)	0 (0.0)	
		Flowerpots (n = 9)	3 (33.3)	*Aedes* spp.
Nilkantha-6	30	Tires (n = 18)	6 (30.0)	*Aedes* spp.
		Drums (n = 6)	0 (0.0)	
		Plastic container (n = 7)	1 (14.3)	*Culex* spp.
		Flowerpots (n = 4)	1 (25.0)	*Aedes* spp.
Total	100	126	21 (16.7)	

## Data Availability

The datasets generated and/or analyzed during the current study are available in the manuscript and the Supplementary Materials.

## Supplementary Materials

The following supporting information can be downloaded at: https://www.mdpi.com/article/10.3390/v16040594/s1, Table S1: Primer used for conventional RT-PCR; Table S2: Demographic and clinical parameters of dengue patients and OFI patients; Table S3: Laboratory parameters of dengue patients and OFI patients.
