# Supplementary material for: Molecular and Entomological Characterization of 2023 Dengue Outbreak in Dhading District, Central Nepal
Source: Viruses. 2024 Apr 12;16(4):594. doi: 10.3390/v16040594 (PMC11053854; doi:10.3390/v16040594)
Supplement: Supplementary file 1 [file viruses-16-00594-s001.zip › viruses-2936417-supplementary.pdf]

Supplementary Tables

Supplementary Table S1. Primer used for conventional RT-PCR

Assay	Identification	Sequences (5' - 3')	Size (base pair)
Conventional RT-PCR	Dengue Consensus primer		
	DC-1, Forward	TCAATATGCTGAAACGCGCG AGAAACCG	511
	DC-2, Reverse	TTGCACCAACAGTCAATGTCT TCAGGTTC	
	Dengue serotype primer		
	D1-Forward	GGACTGCGTATGGAGTTTTG	490
	D1-Reverse	ATGGGTTGTGGCCTAATCAT	
	D2-Forward	GTTCCTCTGCAAACACTCCA	230
	D2-Reverse	GTGTTATTTTGATTTCCTTG	
	D3-Forward	GTGCTTACACAGCCCTATTT	320
	D3-Reverse	CCATTCTCCCAAGCGCCTG	
	D4-Forward	CCATTATGGCTGTGTTGTTT	399
	D4-Reverse	CTTCATCCTGCTTCACTTCT	

Supplementary Table S2. Demographic and clinical parameters of dengue patients and OFI patients

Characteristics	Category	Dengue patients	OFI patients	P-value
**Age**	Child	48 (39.3)	74 (60.6)	**0.046**
	Adult	230 (83.9)	44 (16.1)	
**Gender**	Female	153 (68.3)	71 (31.7)	0.145
	Male	129 (75.0)	43 (25.0)	
**Travel history**	No	80 (79.2)	21 (20.8)	0.684
	Yes	8 (88.9)	1 (11.1)	
**Myalgia**	No	26 (83.9)	5 (16.1)	0.525
	Yes	62 (78.5)	17 (21.5)	
**Rash**	No	59 (72.8)	22 (27.2)	**0.001**
	Yes	29 (100.0)	0 (0.0)	
**Diarrhea**	No	78(78.8)	21 (21.2)	0.690
	Yes	10 (90.9)	1 (9.1)	
**Vomiting**	No	59 (74.7)	20 (25.3)	**0.033**
	Yes	29 (93.5)	2 (6.5)	
**Persistent Vomiting**	No	71 (76.3)	22 (23.7)	**0.022**
	Yes	17 (100.0)	0 (0.0)	
**Retro-orbital pain**	No	36 (65.5)	19 (34.5)	**<0.001**
	Yes	52 (94.5)	3 (4.5)	
**Any bleeding**	No	88 (88.0)	22 (20.0)	-
**Anorexia**	No	32 (78.0)	9 (22.0)	0.693
	Yes	56 (81.2)	13 (18.8)	
**Nausia**	No	42 (71.2)	17 (28.2)	**0.013**
	Yes	46 (90.2)	5 (9.8)	
**Abdominal pain**	No	76 (77.6)	22 (22.4)	0.120
	Yes	12 (100.0)	0 (0.0)	
**Admission status**	No	238 (67.6)	114 (32.4)	**<0.001**
	Yes	44 (100.0)	0 (0.0)	

Supplementary Table S3. Laboratory parameters of dengue patients and OFI patients

Blood parameters	Dengue patients Median (IQR)	OFI patients Median (IQR)	P-value
**Hemoglobin**	12.8 (11.7-14.3)	12.7 (12.0-13.6)	0.420
**Total count (WBC)**	5250 (3628-6630)	6675 (5167-8732)	**<0.001**
**Neutrophils**	68 (56.7-75.0)	62.5 (50.0-71.3)	**0.020**
**Lymphocytes**	21.0 (16.0-31.0)	27.0 (18.8-41.0)	**0.010**
**Eosinophils**	1.0 (1.0-3.0)	2.0 (1.0-3.0)	0.319
**Monocytes**	8.0 (5.0-11.0)	7.0 (5.0-10.0)	0.323
**Platelets**	171.6 (120.0-233.0)	207.0 (150.5-264.0)	**<0.001**
**HCT**	40.5 (36.5-44.3)	39.0 (37.0-42.0)	0.256
**Serum urea**	41.0 (32.3-50.6)	43.0 (34.5-55.0)	0.371
**Creatinine**	1.0 (0.9-1.1)	0.9 (0.8-1.1)	0.346
**Sodium**	136.0 (134.0-138.0)	136.5 (134.0-138.6)	0.791
**Potassium**	3.8 (3.5-4.0)	3.8 (3.5-4.2)	0.357
**SGPT**	42.0 (24.0-64.0)	34.0 (25.0-94.0)	0.422
**SGOT**	41.0 (30.0-78.0)	39.0 (26.0-58.0)	0.564
**ALP**	241.0 (185.0-319.0)	268.0 (200.0-417.0)	0.429
**Bilirubin-Total**	0.6 (0.6-0.8)	0.7 (0.6-0.8)	0.435
**Bilirubin-Direct**	0.15 (0.1-0.2)	0.2 (0.1-0.2)	0.294
